# Supplementary material for: Validating molecular target-enriched fMRI for disentangling drug effects on dopamine
Source: Imaging Neurosci (Camb). 2026 Feb 24;4:IMAG.a.1139. doi: 10.1162/IMAG.a.1139 (PMC12934004; doi:10.1162/IMAG.a.1139)
Supplement: Supplementary Material [file IMAG.a.1139_supp.pdf]

Supplementary information

Supplementary Table 1. List of all clusters with effects of methylphenidate in the models with a single target-enriched regressor at $p < 0.001$ uncorrected and/or $p < 0.05$ with cluster-level FWE correction at the whole-brain level. The x y z coordinates are in MNI152 coordinate space. MPH: methylphenidate; PBO: placebo; DAT: dopamine transporter; NET: noradrenaline transporter; SERT: serotonin transporter.

Target-enriched functional connectivity contrast	Effect direction	x	y	z	Voxel Z-value	Cluster size (k)	p(k) uncorrected	p(k) FWE correction
MPH - PBO: DAT-enriched connectivity	positive	39	-40	62	6.143	1737	4.95e-32	0.00e+00
MPH - PBO: DAT-enriched connectivity	positive	-63	-10	27	4.899	327	5.21e-11	6.25e-10
MPH - PBO: DAT-enriched connectivity	positive	-7	-86	36	4.684	114	8.06e-06	9.67e-05
MPH - PBO: DAT-enriched connectivity	positive	23	-86	39	4.283	64	3.42e-04	0.004
MPH - PBO: DAT-enriched connectivity	negative	-23	-20	-5	4.903	500	2.25e-14	2.70e-13
MPH - PBO: DAT-enriched connectivity x ventral striatal DA synthesis capacity	positive	6	-63	51	4.582	61	3.06e-04	0.004
MPH - PBO: NET-enriched connectivity	negative	39	-17	19	Inf	5362	2.11e-65	0.00e+00
MPH - PBO: NET-enriched connectivity	negative	-37	-20	19	7.531	1266	1.96e-25	0.00e+00
MPH - PBO: NET-enriched connectivity	negative	19	-46	-2	5.763	1161	4.78e-24	0.00e+00
MPH - PBO: NET-enriched connectivity	negative	-7	59	24	5.713	432	8.62e-13	9.96e-12
MPH - PBO: NET-enriched connectivity	negative	-23	-17	-20	5.228	77	1.51e-04	0.002
MPH - PBO: NET-enriched connectivity	negative	-50	30	-5	4.717	146	1.40e-06	1.62e-05
MPH - PBO: NET-enriched connectivity	negative	-7	36	-14	4.555	109	1.52e-05	1.76e-04
MPH - PBO: SERT-enriched connectivity	positive	36	-40	62	5.675	652	3.81e-19	0.00e+00
MPH - PBO: SERT-enriched connectivity	positive	19	-89	36	4.908	82	2.38e-05	3.39e-04
MPH - PBO: SERT-enriched connectivity	positive	-53	26	30	4.685	231	5.99e-10	8.54e-09
MPH - PBO: SERT-enriched connectivity	positive	-23	-73	36	4.467	100	5.28e-06	7.52e-05
MPH - PBO: SERT-enriched connectivity	positive	-53	-10	48	4.351	165	4.27e-08	6.09e-07
MPH - PBO: SERT-enriched connectivity	negative	6	-20	10	5.273	478	1.06e-15	1.51e-14

Supplementary Table 2. List of all clusters with effects of methylphenidate in the model including both DAT and NET target-enriched regressors at p < 0.001 uncorrected and/or p < 0.05 with cluster-level FWE correction at the whole-brain level. The x y z coordinates are in MNI152 coordinate space. MPH: methylphenidate; PBO: placebo; DAT: dopamine transporter; NET: noradrenaline transporter; SERT: serotonin transporter.

Target-enriched functional connectivity contrast	Effect direction	x	y	z	Voxel Z-value	Cluster size (k)	p(k) uncorrected	p(k) FWE correction
MPH - PBO: DAT-enriched connectivity	positive	29	-23	68	5.62	1243	1.74e-25	0.00e+00
MPH - PBO: DAT-enriched connectivity	positive	-40	-13	48	4.51	61	4.80e-04	0.006
MPH - PBO: DAT-enriched connectivity	positive	19	-86	39	4.27	52	0.001	0.012
MPH - PBO: DAT-enriched connectivity	negative	10	-7	7	5.72	509	2.22e-14	2.62e-13
MPH - PBO: DAT-enriched connectivity	negative	6	-30	27	4.46	65	3.45e-04	0.004
MPH - PBO: DAT-enriched connectivity	negative	10	-76	13	4.41	84	7.80e-05	0.001
MPH - PBO: NET-enriched connectivity	positive	-4	-73	48	6.35	985	2.26e-22	0.00e+00
MPH - PBO: NET-enriched connectivity	positive	16	10	68	5.79	642	5.34e-17	6.66e-16
MPH - PBO: NET-enriched connectivity	positive	3	-36	24	5.27	48	0.001	0.016
MPH - PBO: NET-enriched connectivity	positive	3	23	48	5.26	186	7.50e-08	9.11e-07
MPH - PBO: NET-enriched connectivity	positive	-50	13	27	5.14	412	7.82e-13	9.50e-12
MPH - PBO: NET-enriched connectivity	negative	39	-17	19	7.63	497	1.91e-14	2.32e-13
MPH - PBO: NET-enriched connectivity	negative	6	-17	51	6.97	1843	1.35e-33	0.00e+00
MPH - PBO: NET-enriched connectivity	negative	-33	-23	16	6.65	490	2.57e-14	3.12e-13
MPH - PBO: NET-enriched connectivity	negative	26	-17	-17	6.29	119	5.13e-06	6.23e-05
MPH - PBO: NET-enriched connectivity	negative	-23	-17	-17	5.72	543	2.80e-15	3.40e-14
MPH - PBO: NET-enriched connectivity	negative	16	-50	7	5.36	52	0.001	0.011
MPH - PBO: NET-enriched connectivity	negative	-7	59	24	5.19	408	9.37e-13	1.14e-11
MPH - PBO: NET-enriched connectivity	negative	49	-66	7	4.23	39	0.003	0.036
